# The Psycho-Physiological Profile of Adolescent Elite Sailors: Testing a Three-Way Moderation Model

**DOI:** 10.3389/fpsyg.2020.01091

**Published:** 2020-06-18

**Authors:** Anna Antonia Valenzano, Lucia Monacis, Flavio Ceglie, Giovanni Messina, Rita Polito, Maria Sinatra, Giuseppe Cibelli

**Affiliations:** ^1^Department of Clinical and Experimental Medicine, University of Foggia, Foggia, Italy; ^2^Department of Humanities, University of Foggia, Foggia, Italy; ^3^Division of Pathological Anatomy, Department of Emergencies and Organ Transplantation, University of Bari, Bari, Italy; ^4^Department of Educational Sciences, Psychology and Communication, University of Bari, Bari, Italy

**Keywords:** Personality traits, individual differences, salivary cortisol, psycho-physiological profile, adolescent elite sailors

## Abstract

The present study aimed at extending the work on individual differences, in the relationship between personality traits and the cortisol response, by examining the interaction effects of sex and the role category of Italian adolescent elite dinghy sailors. Seventy athletes completed a self-reported questionnaire including socio-demographic data, information about the role played on board (helmsmen or bowmen) and the Big Five Questionnaire-2. Salivary cortisol samples were collected at 30 min after awakening the day before competitions. Main findings from bivariate correlations showed positive associations among cortisol levels, extraversion and consciousness in both male and female bowmen groups. The moderation and moderated moderation analyses further indicated (1) a three-way interaction effect in the relationship between extraversion and salivary cortisol, (2) a marginal significant three-way interaction effect in the relationship between neuroticism and salivary cortisol, and (3) no other personality dimensions were significantly predictive of the outcome variable. Our results provided evidence not only about sex differences, but also about the role played on board by the sailors in the linkages between personality traits and the biomarker of the trait component of HPA axis functioning.

## Introduction

An increasing number of studies investigated the individual differences in psychological and physiological responses to stressors and challenging environments. Among psychological factors, personality constructs, rather than environmental factors, have been proposed as major variables in identifying and impacting on biomarkers of stress-sensitive biological systems, such as the hypothalamus-pituitary adrenal (HPA), as indexed by the cortisol response. Indeed, based on theoretical arguments and research showing the substantial heritability of personality (Heath et al., 1992; Bouchard and McGue, 2003), previous investigations tried to uncover the plausible linkages between personality traits and the trait components of the HPA axis functioning, i.e., basal cortisol, and the state components of HPA, i.e., the cortisol awakening response (CAR), yielding, however, inconclusive results (e.g., Evans et al., 2016).

The personality construct has generally been analyzed by the following psychological models: Model of psychosocial characteristics, Model based on Rumination and Emotional Inhibition, Eysenck’s biopsychological model and the Five Factor Model (FFM; Soliemanifar et al., 2018). The last two models have been conceptualized as trait approaches to personality, focused on bio-physiological correlates. Eysenck’s framework comprises three dimensions: extraversion, neuroticism and psychoticism; while, the FFM identifies five dimensions: neuroticism, extraversion, openness, agreeableness, and conscientiousness (Costa and McCrae, 1992; Hartmann, 2006). Within the psycho-physiological research, neuroticism has been one of the most studied traits. Referring to feelings of vulnerability and the negativity of emotional reactions to social stressors (Lahey, 2009), it was found to be positively, negatively, or not related at all to cortisol. Specifically, no associations were reported between neuroticism and cortisol levels, either measured as a baseline, or as the change after a stressful event (Schommer et al., 1999), as the CAR (Chan et al., 2007; van Santen et al., 2011; Hill et al., 2013), or as the change at noon and later in the afternoon (Ferguson, 2008). Conversely, significant associations were reported between this trait and the HPA functioning, usually measured through the area under the curve, with respect to the increase (AUCI; Zobel et al., 2004), or through the difference in cortisol concentrations between the time of awakening and 30–45 min later (Portella et al., 2005; Mangold et al., 2012).

A further complex matter concerns the effects of sex on HPA functioning, within the above mentioned associations; different sex patterns were reported to be close to significance only in women (DeSoto and Salinas, 2015; Puig-Perez et al., 2016), whereas the absence of sex differences occured after controlling the luteal phase (Kajantie and Phillips, 2006; Poppelaars et al., 2019).

Extroversion, indicating individuals who enjoy being with people and are full of energy, contrary to introverts who are less involved in social activities and tend to keep to themselves, is the second well-investigated trait in relation to HPA functioning, although inconclusive results have been yielded. No association was found between extraversion and the variability in early morning salivary cortisol levels (Munafò et al., 2006; van Santen et al., 2011) and between higher mean scores on extraversion and lower cortisol reactivity to a social stress response in adolescents (Evans et al., 2016). Significant associations were shown between high introversion and low CAR levels among both male and female adolescents, even if no significant associations emerged in the awakening cortisol levels or in the diurnal cortisol slope (Hauner et al., 2008). Conversely, positive associations occurred between higher extraversion and greater CAR levels in females exhibiting greater cortisol output (Hill et al., 2013) or when considering basal cortisol levels in adolescents (Laceulle et al., 2015). Puig-Perez et al. (2016) reported mixed results, from no association when considering the total sample or the 2-Day CAR group, to a negative association when including the data of only one day CAR measurement. With regard to the remaining personality traits of the FFM, no significant associations were found with any kind of cortisol response (Hill et al., 2013).

In light of the inconsistency of these results, the current research sought to examine the individual differences in the relationships between personality traits and the salivary cortisol in adolescent elite sailors. According to the authors’ knowledge, the relevance of this research is that the psycho-physiological profile of dinghy sailors has not yet been empirically analyzed. Only Manzanares Serrano and colleagues distinguished, although theoretically, bowmen’ and helmsmen’ profiles: the former are more extrovert and the latter more determined and introverted with higher levels of self-control (Manzanares Serrano et al., 2012). Consequently, it was expected that the helmsmen should be characterized by higher levels of consciousness and lower levels of openness, agreeableness, neuroticism, and extraversion; whereas, the bowmen should be characterized by lower levels of consciousness and higher levels of openness, agreeableness, neuroticism, and extraversion. Being responsible for maneuvering and handling the boat in all environmental conditions and situations including emergencies and being conscious of the safety of the crew at all times, the helmsmen should tend to be less emotionally instable and more conscious and introverted. Controlling sails, spinnakers, etc., the bowmen tend to be more extroverted, action-oriented, and sociable with the other members of the crew and with the helmsman. Following Kern and Friedman’s assumption that extroverts are characterized by a “biologically-based drive for activity” being oriented to the surrounding environment, bowmen were expected to exhibit elevated cortisol levels, in contrast to the lower cortisol levels of introverted helmsmen (Kern and Friedman, 2011). In addition, as cortisol is the hormone most closely associated with a biological reaction to a stressor, a positive relationship was hypothesized between cortisol and neuroticism, a trait that ought to characterize the bowmen’s profile. Finally, it was supposed thea moderated role of sex in the linkages of cortisol levels with extraversion and neuroticism and a stronger effect of this association in female bowman was supposed. This idea is rooted in the notion that women deal with stressors differently from than men do, including the hormonal levels (Kirschbaum et al., 1992) and their lower coping ability.

## Materials and Methods

### Participants and Procedure

The sample was composed of 70 sailors (48 males and 22 females), with 27 in the under 16 category (15–14 years) and 43 in the under 19 category (16–18 years), all participants of the Italian Youth Two Crew Members Dinghy Classes Championship, held in Bari, Italy, in September 2019. Exclusion criteria were the presence of any form of contraception and a menstrual cycle outside the range of 28 ± 1 days. The cultural and the socioeconomical background of the athletes was homogenous. The research proposal was submitted to the Italian Sailing Federation Committee for approval. In addition, the Regional Committee for Medical and Health Research Ethics approved the study, which was conducted in accordance with the Helsinki declaration. Since most of the athletes were under the legal age of consent, only their attorney/legal representative provided written informed consent for participation. All results were treated anonymously.

### Data Collection

All the data were collected the day before the competitions. Initially, a questionnaire was applied, containing information regarding sailors’ practical experience: dinghy class, role on board, and years of practicing.

### Salivary Cortisol Assay

Saliva collection and cortisol assays were performed as previously described (Capranica et al., 2017). Briefly, the saliva specimens were collected the day before the competitions, within 30 min after awakening, by participants, under parents’ or coaches’ supervision, using cotton swabs and saliva collecting tubes (Salivette, Sarstedt, Germany). The samples were kept in a portable cooler during sampling and then, once returned, were stored at −70°C, until use. A commercially available enzyme immunoassay kit (Salimetrics LLC, State College, PA, United States) was used to analyze salivary cortisol, according to the manufacturer’s instructions.

### Personality Traits

To assess the sailors’ personality characteristics, the Big Five Questionnaire-2 (BFQ-2, Caprara et al., 2007) was used. The BFQ-2 is a phrase-based self-report inventory, comprising 134 items that identify five dimensions (extroversion, agreeableness, consciousness, neuroticism, openness to experience) to describe and assess the personality of adolescents aged over 14. Each dimension included 24 items on a 5-point Likert-type scale, ranging from 1 (*strongly agree*) to 5 (*strongly disagree*). In the current research, alpha coefficients ranged from 0.81 to 0.83.

### Data Analysis

Descriptive statistics and zero-order correlations between the variables of interest were applied to the total sample, sex, and sailor roles on board. Age, sex, roles, and salivary cortisol differences for the variables’ scores were analyzed using independent samples t-tests.

**FIGURE 1:**
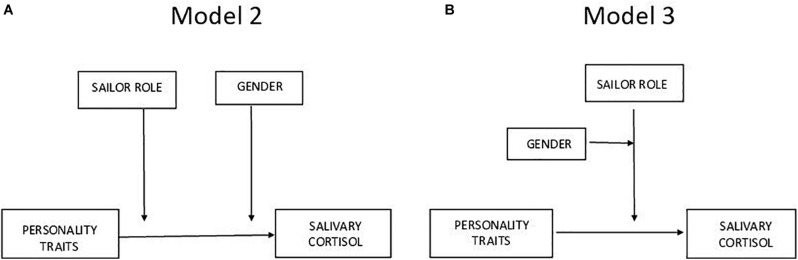
Moderated analyses computed using both moderators, simultaneously **(A)** and the moderated moderation **(B)**.

A multiple regression analysis was performed to identify the best predictors for cortisol levels. The SPSS PROCESS macros version 3.1 (Hayes, 2018) was used for bootstrapping analyses to determine the significance of moderation. The interaction effects of personality traits on cortisol levels, *via* the moderators, were considered significant if 95% bootstrap confidence intervals from 10,000 bootstrap samples did not include zero. Single moderation models were first conducted to test whether the influence of each personality trait (X) on cortisol levels (Y) was moderated by sailing category and sex (W and Z, respectively) (Figure 1). To this purpose, moderated analyses were computed using both moderators, simultaneously (Model 2, Figure 1A) and the moderated moderation (Model 3, Figure 1B).

## Results

The physical and technical variables analyzed in this study are shown in Table 1. It is worth noting that the number of male sailors exceeded that of female sailors, either in the total sample, or in each category, while, with regard to the role held on board, the helmsmen were slightly more numerous than the bowmen. No differences were found in the years of practicing, either between male and female sailors, or between helmsmen and bowmen, confirming that this variable did not influence the result.

**TABLE 1:** Descriptive statistics: Mean and standard deviation for each variable in the total sample, male and female group, helmsmen and bowmen group.

	Total sample	Male group	Female group	Helmsmen	Bowmen

	*n* = 70	*n* = 48	*n* = 27	*n* = 37	*n* = 33

Mean (SD)					
Age	15.95 (1.28)	15.74 (1.20)	16.38 (1.39)	15.84 (1.38)	16.13 (1.13)
C (μg/dl)	3.93 (1.24)	4.14 (1.07)	3.49 (1.48)	3.86 (1.11)	4.50 (1.37)
E	82.97 (12.63)	83.22 (12.61)	82.46 (13.16)	83.96 (12.88)	81.33 (12.45)
A	84.73 (11.81)	84.37 (10.81)	85.46 (14.12)	84.56 (10.24)	85.00 (14.44)
C	82.65 (11.22)	81.15 (9.97)	85.77 (13.36)	81.20 (11.20)	85.07 (11.22)
N	65.88 (14.64)	68.52 (14.77)	60.38 (13.23)	64.16 (14.20)	68.73 (15.42)
O	81.22 (12.67)	78.48 (12.15)	88.92 (12.24)	80.40 (12.05)	82.60 (13.98)

*C (μg/dl), cortisol; E, extroversion; A, agreeableness; C, consciousness; N, neuroticism; O, openness to experience.*

The impact of athletes’ personality on the biological trait models of stress reactivity was indexed by cortisol reactivity. The total and detailed awakening cortisol analyses are shown in Table 2. The results showed that there were no differences among athletes in the cortisol awakening levels, measured the day before competition, in relation to age, sex, and role on board, even though bowmen showed an increasing trend in cortisol reactivity, compared to helmsmen (*p* = 0.068), which did not reach significance, probably because of the small sample size (*n* = 70) included in the analysis.

**TABLE 2:** Bivariate correlations between personality traits and levels of cortisol in the total sample, sex group, sailing group, and the sailing role within sex groups.

	Total Sample	M	F	H	B	H		B	

Cortisol levels									
						M	F	M	F
E	0.200	0.080	0.381	0.010	0.516*	0.175	−0.275	0.160	0.920*
A	0.173	0.074	0.300	0.051	0.269	−0.131	0.288	0.302	0.361
C	0.266	0.352	0.307	−0.105	0.583*	0.058	−0.251	0.201	0.745
N	0.164	−0.025	−0.341	−0.153	0.430	−0.318	−0.046	−0.064	0.797
O	0.226	0.245	0.427	0.155	0.266	0.206	0.197	0.236	0.655

*p < 0.05. E, extroversion; A, agreeableness; C, consciousness; N, neuroticism; O, openness to experience; M, males; F, females; H, Helmsmen; B, Bowmen.

With regard to the total sample, sex differences emerged only in the mean scores of openness to experience, *t*(68) = −2.053, *p* = 0.047, where females obtained higher mean scores (*M* = 88.92) in comparison to males (78.48). On the other hand, when considering the role, sex effects emerged in the mean scores of consciousness in helmsmen, *t*(68) = −2.568, *p* = 0.017, a category in which females obtained higher mean scores (*M* = 88.75), compared to males (*M* = 77.61).

Moreover, sailing role effects were also examined in the total sample. Significant differences emerged in the mean scores of cortisol levels, *t*(68) = −2.065, *p* = 0.046, among sailors. Bowmen obtained higher mean scores (*M* = 4.50) in comparison to helmsmen (*M* = 3.86). When considering this effect within sex groups, significant differences were observed in the mean scores of cortisol levels, *t*(35) = −3.565, *p* = 0.002, and consciousness, *t*(35) = −2.638, *p* = 0.014: male bowmen obtained higher scores compared to male helmsmen in both variables of interest (*M* = 4.93 and 3.67, *M* = 87.10 and 77.85, respectively). No significant differences emerged within female bowmen and helmsmen.

Table 2 provides a first picture of the interrelationships between cortisol levels and personality traits in the total sample, male and female groups, and helmsmen and bowmen. No significant association emerged between cortisol levels and personality traits in the total sample and in the sex groups, whereas positive associations between cortisol levels, extraversion, and consciousness in the bowmen group were shown. Furthermore, a positive association between levels of cortisol and extraversion was also confirmed in the female bowmen group. A series of moderation analyses and moderated moderation analyses were performed to examine whether the relationship between personality traits and cortisol levels was influenced by sailing role and sex, and how these moderators changed the strength (stronger or weaker) of the linkage in predicting the levels of salivary cortisol. To this purpose, sailing role and sex were computed simultaneously (Model 2). It was further examined whether the effect of personality traits on the cortisol response was a function of the conditional effects between personality traits and sailing role by sex (X × W × Z) (Model 3).

**FIGURE 2:**
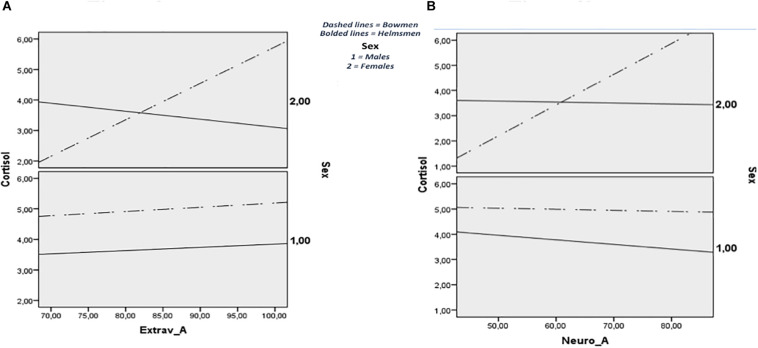
A three-way interaction effect, Extraversion **(A)**, Neuroticism **(B)**.

Table 3 shows the results of the analyses in both models. With regard to the relation of extraversion with salivary cortisol levels, Model 2 indicated no statistical interaction effects of both moderators. Conversely, Model 3 showed a significant three-way interaction effect and is plotted in Figure 2A. When looking at the regression coefficient for XWZ, i.e., *b* = 0.142, *t*(32) = 2.596, *p* = 0.014 with a 95% CI of 0.031 to 0.252, the magnitude of the moderation by sailing role of the effect of extraversion on cortisol, depended on the sex category. The effect of extraversion on cortisol was positive, but the difference of this effect between helmsmen and bowmen was stronger in female bowmen. This moderated moderation accounted for 10% of the variance in support of the cortisol levels. With regard to the traits of agreeableness, consciousness, and openness to experience, both models indicated no significant direct effect or interaction effect in the relationship with cortisol levels, whereas the association between neuroticism and cortisol levels was moderated by sailing role (Model 2; *b* = 0.05, *t*(34) = 2.204, *p* = 0.034 with a 95% CI of 0.004 to 0.105) and by the product of the two moderators (Model 3; *b* = −8.054, t(32) = −2.228, *p* = 0.033 with a 95% CI of -1.419 to -0.690). Finally, in this relationship a three-way interaction effect was close to being significant (Model 3; *b* = 0.112, *t*(32) = 1.998, *p* = 0.0543 with a 95% CI of -0.002 to.226), thus indicating a slight significant moderated moderation of sailing role, by sex category. The positive relationship between neuroticism and salivary cortisol tended to be stronger in female bowmen. The three-way interaction is plotted in Figure 2B.

**TABLE 3:** Unstandardized beta coefficients and model indices.

Predictors	Model 2 *R* = 0.568 *R*^2^ = 0.323, *p* = 0.016	Model 3 *R* = 0.696 *R*^2^ = 0.485, *p* = 0.001
E	−0.091	0.019
Role	−3.870	13.975 (*p* < 0.05)
Sex	−2.794	15.90 (*p* < 0.05)
E × Role	0.058	−0.132
E × Sex	0.026	−0.179 (*p* < 0.05)
Role × Sex		−12.956 (*p* < 0.01)
E × Role × Sex		0.142 (*p* < 0.05)

	**Model 2 *R* = 0.567 *R*^2^ = 0.322, *p* = 0.017**	**Model 3 *R* = 0.651 *R*^2^ = 0.424, *p* = 0.008**

N	−0.138 (*p* < 0.05)	0.066
Role	−2.789	8.431
Sex	−3.774 (*p* < 0.05)	6.954
N × Role	0.055 (*p* < 0.05)	−0.098
N × Sex	0.052	−0.097
Role × Sex		−8.054 (*p* < 0.05)
N × Role × Sex		0.112 (*p* = 0.054)

*E, Extroversion; N, Neuroticism.*

## Discussion

The current study examined the individual differences in the relationships between personality traits and the trait components of HPA axis functioning, i.e., the awakening cortisol, in a sample of adolescent elite sailors. Moreover, given the lack of empirical evidence for the different psychological profiles of helmsmen and bowmen, this investigation sought to fill this gap by exploring whether the different roles played by these athletes could moderate the linkages between personality traits and awakening cortisol levels. It was also explored whether sex could influence the moderated associations. With regard to the trait components of HPA axis functioning, we found evidence that cortisol awakening levels were not significantly influenced by the physiological response of the training load imposed on the athletes, with respect to age, sex, and years of practicing. Notably, we might speculate that cortisol awakening levels are positively influenced by the role played on board. However, these findings posit the possibility that such a different response detected in both helmsmen and crew members were consistent with the different training loads, related to the role. Our findings further corroborated those studies that showed significant relationships of awakening cortisol levels with extraversion (Schommer et al., 1999; Oswald et al., 2006; Hauner et al., 2008; van Santen et al., 2011; Hill et al., 2013) and neuroticism (Zobel et al., 2004; Portella et al., 2005; Laceulle et al., 2015). Interestingly, descriptive statistics indicated sex effects in the sailor category, that is, female helmsmen tended to be more conscious in comparison to their counterparts. This result not only confirmed the above-mentioned hypothesis of helmsmen’ tendency to obtain higher scores for consciousness, as this role is related to the responsibility of the safety of crew and boat, but it also shed light on sex differences in sailing roles. In this vein, the bio-psychological linkage between sex and personality characteristics could suggest to coaches how sailor roles should be selected. When considering the biomarker factor, the obtained higher mean scores of salivary cortisol in bowmen could be explained properly by the sailing role, given bowmen’s tendency to be more action-oriented and to show higher cortisol levels. However, an unexpected result was the sailor role effect on consciousness; in fact, compared to male helmsmen, male bowmen obtained higher scores on this trait, which was contrary to the hypothesized assumption. A possible explanation may be inferred from a lacking psychological assessment of the specific profiles during athletes’ selection.

In alignment with some other studies (Hauner et al., 2008; van Santen et al., 2011; Hill et al., 2013; Laceulle et al., 2015), extraversion was significantly associated with cortisol levels. Specifically, the positive association that supported Kern and Friedman’s idea of the biological drive for activity, emerged in the bowmen category (Kern and Friedman, 2011). When stratified by sex, the same association was confirmed only in female bowmen, consistent with previous research (Kunz-Ebrecht et al., 2004; Wright and Steptoe, 2005; Almeida et al., 2009) and with the assumption that females are more likely to report chronic stress than males (McDonough and Walters, 2001), which may impact neuroendocrine functioning (Pruessner et al., 1997; Wüst et al., 2000). The three-way interaction observed in the moderated moderation further proved these sex differences in the HPA system.

When looking at neuroticism, findings corroborated the significant trend of elevated cortisol levels, that is, higher levels of neuroticism were positively associated with higher baseline levels of cortisol (Portella et al., 2005; Nater et al., 2010; Oishi et al., 2012; Garcia-Banda et al., 2014; Miller et al., 2016). Such a trend was consistent with the assumption that individuals high in this trait tend to have an increased magnitude of cortisol secretion during the day, reflecting greater frequency and intensity of HPA stimulation from the psychosocial domain. Following the suggestion to check whether males and females differ in HPA activation in the neuroticism-cortisol relationship (DeSoto and Salinas, 2015), a further aim of the current research was to examine the key role of sex. Results provided the existence of a gender specific interaction: neuroticism and cortisol levels were noted to be positively related among females. This finding was in line with Puig-Perez and colleagues’ investigation, but in contrast to Oswald’s research group and DeSoto and Salinas, who found negative relationships, and with Zobel et al., too, who reported a positive association among males (Zobel et al., 2004; Oswald et al., 2006; DeSoto and Salinas, 2015; Puig-Perez et al., 2016).

Other important information from the current research, concerned the hypothesized positive relationship between cortisol and neuroticism, which was yielded in the bowmen’s category. As such, the empirical evidence took a first step in addressing sport research (e.g., Frenkel et al., 2019) in the examination of the dispositional psychological factors in stressful situations before a competition. Despite this strength, our study was not without limitations. The sample size was modest (but sufficient for the analysis carried out) and the cortisol survey pertained only a single awakening time, without taking into account cortisol changes in response to a stressor or individual differences in diurnal variations. Consequently, the comparability of the current findings to prior findings were, therefore, restricted. Further work should be carried out to clarify the existing inconsistent data and to generalize the present findings.

## Conclusion

The current research examined the psycho-physiological profile of adolescent elite sailors by focusing on individual differences in the relationships between personality traits and the salivary cortisol response. To this purpose, two models were tested: in the former model sailing role and sex were computed simultaneously when considering the linkages between personality traits and cortisol levels; whereas, in the latter model, the function of the conditional effects between personality traits and sailing role by sex was taken into account when considering the effect of personality traits on cortisol response. In summary, these findings suggested that the effect of extraversion on cortisol was positive in female bowmen. Likewise, the effect of neuroticism on cortisol tended to be marginally significant and positive in female bowmen. No other personality traits were significantly predictive of cortisol levels. Therefore, our results extended the knowledge on previous contrasting findings, shedding light on the importance of sex differences and the role of adolescent dinghy sailors, when examining the relationships between personality trait and the HPA system.

## Data Availability Statement

The datasets generated for this study are available on request to the corresponding author.

## Ethics Statement

The studies involving human participants were reviewed and approved by Regional Committees for Medical and Health Research Ethics. Written informed consent to participate in this study was provided by the participants’ legal guardian/next of kin.

## Author Contributions

GC and LM: conceptualization, LM: methodology and formal analysis. FC, GM, AV, and RP: investigation. LM and MS: writing-original draft preparation. LM and MS: writing-review and editing. MS: supervision. All authors contributed to the article and approved the submitted version.

## Conflict of Interest

The authors declare that the research was conducted in the absence of any commercial or financial relationships that could be construed as a potential conflict of interest.
